# Exploring the Health Benefits and Therapeutic Potential of Roselle (Hibiscus sabdariffa) in Human Studies: A Comprehensive Review

**DOI:** 10.7759/cureus.49309

**Published:** 2023-11-23

**Authors:** Ali Almajid, Ali Bazroon, Alzahraa AlAhmed, Omar Bakhurji

**Affiliations:** 1 Internal Medicine, King Fahad Specialist Hospital, Dammam, SAU; 2 Faculty of Medicine, King Abdulaziz University Hospital, Jeddah, SAU; 3 Medicine, Imam Abdulrahman Bin Faisal University, Dammam, SAU

**Keywords:** roselle, bioactive compounds, health benefits, clinical trials, calyx, hibiscus sabdariffa extracts

## Abstract

Hibiscus sabdariffa (HS), commonly known as Roselle, has a rich history of traditional uses and is recognized for its diverse pharmacological properties, including antihypertensive, anti-inflammatory, antimicrobial, and more. This comprehensive review synthesizes the existing literature on the health benefits associated with the consumption of HS or its ingredients. Key areas of focus include metabolic health, blood sugar, and lipid regulation, wherein studies have reported varying effects on parameters such as fasting blood glucose, insulin sensitivity, and lipid profiles. Furthermore, Roselle exhibits anti-inflammatory properties, as evidenced by its impact on inflammatory markers such as MCP-1 and TNF-α. Additionally, HS extracts have shown notable antibacterial efficacy against various strains, with a potential role in urinary tract infection management. Studies also suggest potential benefits for renal function, with improvements in markers such as blood urea nitrogen and creatinine levels. In this article, we aim to review the existing literature on the health benefits of HS.

## Introduction and background

Roselle, also known as jamaica (in Spanish), red sorrel (in English), or karkadeh (in Arabic), is a plant named Hibiscus sabdariffa (HS), native to India and Malaysia, is a tropical species extensively cultivated in tropical and subtropical regions worldwide, including Central and West Africa as well as South‐East Asia. It goes by various names, such as karkade, bissap, sour tea, and red tea [1-4]. Scientific evidence has demonstrated the pharmacological potential of Roselle, including its antihypertensive, anti-hyperlipidaemic, anti‐inflammatory, antimicrobial, diuretic, uricosuric, and anaemia-treating effects [1,3-6]. It has a history of traditional uses in both culinary practices (in foods, food coloring, and beverages) and as a remedy for various health conditions. For instance, in regions such as China and Thailand, different parts of the Roselle plant (including the flower, leaves, calyx, and corolla) are commonly consumed as a thirst‐quenching beverage. The administration of chemical-based medications is associated with inherent side effects and specific limitations. Conversely, the adoption of herbal remedies may represent a viable option, characterized by a reduced incidence of adverse effects, provided that their application adheres to precise dosages and protocols. This review aims to provide a comprehensive summary of the current literature pertaining to the health benefits associated with HS.

## Review

Cardiovascular health and blood pressure

Hypertension is recognized as a leading cause of cardiovascular morbidity and mortality around the globe. Additionally, hypertension is one of the main modifiable risk factors for the development of end-organ complications, such as myocardial ischemia, cerebrovascular diseases, and renal failure [7]. The main anti-hypertensive drugs used in evidence-based medicine include renin-angiotensin system inhibitors (e.g., lisinopril, captopril), calcium channel blockers (e.g., amlodipine, nifedipine), diuretics (e.g., hydrochlorothiazide (HCTZ), spironolactone), and vasodilators (e.g., hydralazine, minoxidil) [8]. Several biological mechanisms have been proposed to contribute to the anti-hypertensive action of HS extracts (HSE). In a study involving a single male participant with hypertension, who initially had a blood pressure of 180/120 mmHg and had not taken any prior anti-hypertensive medication at baseline, it was found that consuming a single serving of HS tea resulted in a reduction of both systolic and diastolic blood pressure to 150/100 mmHg [9]. These changes were attributed to the relaxing effects of HS. Similarly, in another study involving healthy female participants, after 48 days of intervention, the twice-daily consumption of HS tea at a dose of 2 g in hot water led to a decrease in both systolic and diastolic blood pressure [10].

Ritonga et al. conducted a quasi-experimental study employing a non-equivalent control group design, which demonstrated that the administration of HSE at a dose of 10 g of powder brewed with hot water augmented the anti-hypertensive effects of prescribed medications [11]. In pre-eclampsia, the ingestion of HSE in tablet form three hours after consumption led to a reduction in systolic and diastolic blood pressure among postpartum mothers. It is postulated that the bioactive compounds, particularly flavonoids, present in HS, may lead to the activation of endothelium-driven relaxing factors, facilitating vasodilation and inhibiting the formation of angiotensin-converting enzyme-II (ACE-II) [12].

In a non-randomized quasi-experimental study conducted by Al-Shafei et al. [13], it was observed that regular consumption of HS infusion, in the form of four standard cups, each containing 250 mL with HS calyx, for 28 days, led to a reduction in pulse pressure of up to 22% and a decrease in heart rate from 70 to 58 beats per minute among participants moderately hypertensive compared to the initial readings and the normotensive group [13]. Conversely, discontinuing HS intake resulted in an increase in pulse pressure and heart rate in hypertensive participants. In a sequential randomized controlled study carried out by Haji-Faraji and Tarkhani, which involved 54 individuals with moderate hypertension and an average age of 51 ± 10 years, it was observed that, after two weeks of daily consumption of HS tea, both systolic and diastolic blood pressure showed a significant decrease compared to the control group, by 11.2% and 10.7%, respectively [14]. However, after discontinuing HS tea, the blood pressure returned to the pre-treatment baseline levels. Therefore, regular intake of HS might lead to a gradual reduction and regulation of arterial pressure and heart rate without inducing hypotension. This effect can be attributed to the potential of HS bioactive compounds to modulate mechanisms similar to those activated by histamine, thereby promoting vasorelaxation and vasodilation. Specifically, the inhibition of calcium influx into vascular smooth muscle cells [13].

A comparative study investigated the efficacy of HS intake vs HCTZ on blood pressure and electrolytes in 80 subjects newly diagnosed with mild-to-moderate hypertension. The results showed that the HS group had a greater reduction in systolic and diastolic blood pressure (11.38% and 12.13%, respectively), compared to the HCTZ group (8.55% and 9.59%, respectively). Both treatments caused a reduction in serum sodium, but the HS group had sustained sodium reduction even after treatment withdrawal. On the other hand, HCTZ caused more reduction in serum potassium and chloride, which could potentially pose a risk of electrolyte imbalance in this group. In the end, the authors suggested that increased urinary sodium excretion could be another mechanism of anti-hypertensive effect [15]. In contrast, Herrera-Arellano et al. [5] carried out a randomized controlled trial with stage 1 hypertensive patients, assigning them to either an HS infusion group or a group receiving 50 mg of captopril daily, both for a total duration of four weeks. The results revealed no substantial distinction between the two groups in terms of therapeutic efficacy and tolerability. Interestingly, the authors noted a diuretic effect akin to that of spironolactone in the HS group [5].

In a double-blind, randomized, placebo-controlled study involving a group of healthy individuals aged 18-35 years, Kafeshani et al. found that consuming HS tea daily (450 mg, with 250 mg of anthocyanins) for 42 days led to a notable decrease in systolic blood pressure. However, there was no statistically significant change in diastolic blood pressure compared to the control group [16]. The observed effect is linked to the nitric oxide release from the endothelium, which in turn inhibited calcium influx into vascular smooth muscle cells. Herrera-Arellano et al. conducted a randomized, double-blind study comparing the clinical effects of HS decoction, which contains 250 mg of anthocyanins, with lisinopril in hypertensive participants aged 25-61 years of both genders. After 28 days of daily consumption, participants experienced a reduction in blood pressure from 146/97 to 129/85 mmHg, with no notable adverse events. However, the therapeutic impact of HS was slightly less effective than that of a 10 mg dose of lisinopril (65% effectiveness compared to 82%). These findings were attributed to anthocyanins-mediated inhibition of ACE [17].

In a randomized controlled trial conducted by Nwachukwu et al., it was shown that administering HSE at a dose of 150 mg per kg per day for four weeks led to a 32% reduction in plasma aldosterone levels in newly diagnosed hypertensive individuals not on anti-hypertensive therapy, aged 31-70 years. This reduction was comparable to the effect observed in the lisinopril group (30.01%). Blood pressure levels normalized in 76% of participants in the HS group, as compared to only 65% in the lisinopril group. Furthermore, no reported adverse effects related to HS consumption. In this context, HSE may alleviate hypertension through the inhibition of the renin-angiotensin-aldosterone system mediated by the bioactive properties of anthocyanins [18].

Elkafrawy et al. conducted a phase II, randomized, double-blind study comparing the anti-hypertensive effects of capsules containing standardized HSE (300 mg) and Olea Europea (HS-OE) at a dose of 200 mg, with captopril as the control. The study involved individuals with hypertension grade 1 aged 25-60 years. The results showed that consuming HS-OE capsules for 56 days decreased blood pressure in a similar fashion and effectiveness compared to captopril at a dose of 25 mg. Additionally, a significant percentage of patients, 74%, achieved the desired blood pressure, < 140/90 mmHg, within the intervention group. No clinically significant adverse effects were reported. The observed anti-hypertensive effects were attributed to the phytochemical constituents that mediate ACE inhibition [19].

In a recent exploratory intervention study, Al-Anbaki et al. investigated the impact of decocted HS calyx consumption at a dosage of 10 g in 500 mL of boiling water among individuals with hypertension whose blood pressure was not within target levels. The study, conducted over a six-week period, revealed that 61.8% of participants achieved the recommended blood pressure target of <140/90 mmHg. This favorable outcome is ascribed to the bioactive constituents, namely, anthocyanins and hibiscus acid, present in the HS powder, which demonstrated efficacy through the inhibition of ACE and vasodilatory properties [20]. Parallel observations were documented in a precedent multicenter clinical trial encompassing patients with uncontrolled hypertension whose blood pressure was not within target levels. Notably, 38% of subjects achieved the target blood pressure following four weeks of daily HS decoction consumption at a concentration of 10 g in 500 mL of boiling water. Additionally, the remaining participants experienced a noteworthy reduction in both systolic and diastolic blood pressure values, 10 mmHg [21]. HS exhibits potential in treating hypertension, both in conjunction with or independent of medication [20-21]. Additionally, it has been reported that an HS drink, administered at a dose of 7.5 g of HS calyx powder, demonstrated an acute effect with a reduction in postprandial systolic and diastolic blood pressure in subjects with a cardiovascular risk of ≤ 10%. This reduction was observed four hours after consumption, as compared to the baseline values, in a randomized, cross-over study. These effects were associated with the phenolic compounds of the HS infusion [22].

On the contrary, a randomized controlled trial involving 35 participants diagnosed with metabolic syndrome revealed that the ingestion of HS capsules at a dose of 500 mg of powder, with a concentration of 6 mg per g of anthocyanins, once daily with meals for a duration of four weeks, caused a significant reduction in systolic blood pressure [23]. Similarly, Elawad-Ahmed et al. conducted a prospective cohort study involving 19 adult patients with hypertension to evaluate the effects of HS drink consumption at a dosage of 1,250 mg in 300 mL of water, administered twice daily for a four-week duration. No significant alterations were observed in the parameters examined, specifically blood pressure, blood glucose, lipid profile, and the inflammatory marker C-reactive protein. The authors also suggested that the distinctive characteristics of the study population might exert a decisive influence on these outcomes [24].

In addition, Marhuenda et al. conducted a randomized, double-blind, placebo-controlled study with 80 participants who were either pre-hypertensive or had type 1 hypertension (aged 18-65 years), not receiving pharmacological treatment, and had baseline blood pressure readings of ≥ 120/80 mmHg [25]. They investigated the efficacy of an extract combining HS with Lippia citriodora (LC). The daily oral administration of a tablet containing 325 mg of LC and 175 mg of HS, as a single unit with a total combination dosage of 500 mg, over a 12-week period, led to a progressive reduction in systolic blood pressure. However, no significant effect was observed in diastolic blood pressure. These outcomes were attributed to the specific bioactive compounds present in the plant extracts, including anthocyanins and phenylpropanoids, in terms of both type and concentration.

The anti-hypertensive effect of HS is primarily mediated through processes such as diuresis, vasodilation, regulation of calcium influx, ACE inhibition, and blocking of AT1 receptors [15]. Given this information, HS emerges as a natural, readily available, cost-efficient, and uncomplicated option for managing blood pressure in individuals with mild-to-moderate hypertension, without any reported adverse effects.

Metabolic health, blood glucose, and lipid profile

Diabetes mellitus is a complex endocrine and metabolic disorder marked by persistent hyperglycemia, disturbances in lipid metabolism, and alterations in protein metabolism. These abnormalities arise from impairments in the regulation of insulin secretion and/or its effectiveness in action [26]. Metabolic syndrome encompasses a constellation of metabolic irregularities, encompassing hypertension, central obesity, insulin resistance, and atherogenic dyslipidemia [27]. A clinical trial comprising 100 patients investigated the impact of HS sour tea on insulin sensitivity and fasting blood glucose (FBG). The study's findings indicated that there was no statistically significant reduction in FBG. Curiously, FBG levels were higher at the conclusion of the study. Additionally, the group administered with sour tea displayed higher insulin resistance, denoted by diminished insulin sensitivity (P < 0.001) [28]. On the contrary, pooled statistics from a systematic review and meta-analysis, encompassing eight trials, revealed a significant reduction in serum glucose levels (weighted mean difference = -3.964 mg/dL; 95% confidence interval: -6.227 to -1.702; P = 0.001) when compared to the placebo group [29]. Other studies have also examined the impact of HS on blood glucose profiles, as indicated in Table 1. In general, HS demonstrates the capacity to lower blood glucose levels in individuals who are healthy, pre-diabetic, or have diabetes. This effect is primarily attributed to its phytochemical constituents, which enable it to regulate the digestion of carbohydrates and enhance insulin secretion and/or sensitivity.

**Table 1:** Effect of HS on blood glucose COT: cross-over trial; RCT: randomized-controlled trial; DM: diabetes mellitus; QE: quasi-experimental

Design	Sample size	Participants	Intervention	Control	Duration	Outcome	Ref.
COT	22	Male with 10-year CVD risk ranging from 1-10% and no history of smoking or chronic illnesses	7.5 g HS beverage	Yes	4 hours	inclination towards a reduced postprandial insulin response compared to the control group.	(22)
RCT	52	Subjects with Type 2 DM	HS 500 mg tablet/capsule 2 times daily	Yes	8 weeks	HS significantly reduced fasting blood glucose and insulin levels	(46)
QE	24	Women with pre-diabetes	HS-stevia tea twice a day. 5 g HS and 125 mg Stevia sweetener	Yes	2 weeks	HS significantly decreased fasting blood glucose levels but not 2-hour post-prandial glucose	(47)

A double-blind placebo-controlled trial conducted by Asgary et al. enrolled 40 patients with metabolic syndrome and found that 500 mg/day of HS calyx powder causes a significant reduction in triglyceride levels at week four. Triglyceride levels at week four were 187 vs 269 mg/dL in the experimental and control groups, respectively (P = 0.044). However, no significant effect was noted among markers of inflammation, oxidative stress, and blood glucose level [23]. On the contrary, another trial conducted by Gurrola-Díaz et al. looked at patients with metabolic syndrome and found that 100 mg/day of HSE powder reduced blood glucose and total cholesterol levels and increased high-density lipoprotein HDL levels [30]. The observed disparities in outcomes among the aforementioned studies can likely be attributed to variations in the concentration of polyphenols and other constituents within HS, specifically between its powdered and extract forms. Notably, the extract powder employed in the trial conducted by Gurrola-Díaz et al. exhibited a substantially higher concentration of polyphenols and other constituents compared to the whole powdered form of HS. Another study conducted by Elkafrawy et al. found a significant reduction in triglycerides levels at eight weeks with a low dose, fixed combination of Roselle powder and olive leaves' powder taken twice daily (P = 0.038) [19]. However, higher doses of the same combination did not achieve a significant reduction in triglyceride levels, suggesting a dose-dependent effect of combined Roselle powder and olive leaves’ powder on triglyceride levels. In contrast, a meta-analysis conducted by Zhang et al., incorporating nine trials, yielded results that did not exhibit a statistically significant impact on triglyceride levels (weighted mean difference = -0.77; 95% confidence interval (-7.87, 6.33); P = 0.832) [31].

Dyslipidemia is widely recognized as a significant risk factor for the development of coronary heart disease and various associated health complications [32]. Low-density lipoprotein (LDL) cholesterol is associated with an increased risk of cardiovascular disease and is associated with diabetes, hypertension, hypertriglyceridemia, and atherosclerosis [33-34]. Oxidized LDL drives the initial process of early atherosclerosis [35-36]. The antioxidant potential of protocatechuic acid (PCA), a phenolic compound isolated from the dried flower of HS, and esculetin (ECT) in inhibiting LDL oxidation was evaluated by Lee et al. [37]. The findings demonstrate that both PCA and ECT exhibit inhibitory activity against LDL oxidation. When compared to other antioxidants, PCA and ECT show similar effects on LDL oxidation at equivalent concentrations to curcumin or vitamin C, but exceed those of vitamin E. Although the precise mechanism is not fully understood, some researchers suggest that ECT and PCA may cause a reduction in the generation of free radicals, thereby protecting LDL by impeding the formation of lipid peroxides [37]. The study by Lin et al. investigated the impact of three concentrations of HSE on human cholesterol levels, which ranged from 175 to 327 mg/dL, over a duration of four weeks. The administered capsules contained 500 mg of pre-standardized HSE, consisting of anthocyanins (20.1 mg), flavonoids (10 mg), and polyphenols (14 mg) [38]. Different daily doses of HSE were examined, with participants divided into three groups: 1.5 g, 3 g, and 4.5 g. Baseline levels of cholesterol were measured at two and four weeks. The findings demonstrated a notable reduction in cholesterol levels across all three groups, averaging 2.6%, 4.4%, and 2.5% by the fourth week, respectively. The outcome was consistently observed in both the second and fourth weeks, and the best response to the treatment was at the 3 g dose. A meta-analysis incorporated nine trials comprising a total of 503 participants indicated that, in comparison to the control group, supplementation with HS led to a significant reduction in total cholesterol (weighted mean difference = -14.66; 95% confidence interval (-18.22, -11.10); P = 0.000) and LDL cholesterol (weighted mean difference = -9.46; 95% confidence interval (-14.93,-3.99); P = 0.001) [31].

Increased body mass index (BMI) is associated with various diseases, including pulmonary, gastrointestinal, liver, cardiovascular, diabetes mellitus and insulin resistance, metabolic, and renal diseases [39-43]. Asgary et al. found a significant reduction in the BMI among 20 patients receiving 500 mg/day HSE powder where a BMI at baseline and at four weeks was 27.51 vs 27.27 kg/m^2^ in the experimental group, yet no statistical significance noted among the placebo and experimental group [23]. While the detected difference in BMI between the baseline and the four-week assessment is marginal, it may have attained greater clinical relevance had participants been subjected to higher concentrations of HS over an extended period, or if the study encompassed a larger sample size.

The consumption of complete HS beverage (HSB) may confer potential health benefits. An investigation assessed the impact of HSB on a group of 30 well individuals of Asian descent. Each participant was provided with 200 mL of HSB in both morning and evening for a consecutive period of 30 days. Blood samples were collected to assess lipid profile, and measurements for height, weight, and abdominal circumference were recorded at baseline and upon completion of the 30-day period. The analysis revealed a statistically significant decrease in abdominal circumference along with an elevation in HDL cholesterol levels, between the initial and final assessments. Furthermore, the study demonstrated an adverse impact on LDL cholesterol levels, which exhibited an increase, and a favorable effect on triglyceride levels, which were lower at the 30-day mark compared to the baseline. It is important to note, however, that these changes did not achieve statistical significance [44]. Another study, as reported by Mozaffari-Khosravi et al., demonstrated that patients who ingested 240 mL of tea infused with a 2 g sachet of Roselle twice daily, for a duration of one month, experienced a significant elevation in HDL levels. Concurrently, there was a significant reduction in the levels of total cholesterol, LDL, triglycerides, and Apo-B100 in the bloodstream compared to their pre-treatment levels [45]. Other studies have also examined the impact of HS on blood glucose and lipid profiles, as indicated in Tables 1-2, respectively [22,46-53].

**Table 2:** Effect of HS on lipid profiles NI: no information; DM: diabetes mellitus; LDL: low-density lipoprotein; HDL: high-density lipoprotein; RCT: randomized-controlled trials; DBT: double-blind trial; CT: controlled trial; NRCT: non-randomized controlled trial; MES: metabolic syndrome; TC: total cholesterol; TG: triglyceride; RT: randomized trial; NNT: non-randomized, non-controlled trial; OxLDL: oxidized low-density lipoprotein

Design	Sample size	Participants	Intervention	Control	Duration	Outcome	Ref.
NI	32	Excluded if history of smoking, DM, or taking any medications	HS beverage 35 g	No	2 weeks	Reduced the total cholesterol and LDL and increased HDL	(48)
RCT	57	Subjects without chronic illnesses and baseline LDL 130-190 mg/dL	1 g HS capsule/tablet	Yes	3 months	10% reduction in triglyceride values in the experimental arm while the placebo group showed no significant change	(49)
RCT	36	Subjects with obesity	450 mg HS capsule/tablet	Yes	3 months	No significant changes in HDL and LDL levels	(50)
NRCT	18	Subjects with MES	2 g twice daily HS tea	Yes	21 days	HS reduced TC, TG, HDL, and LDL in the experimental group	(51)
RCT	84	Subjects with hypertension	15 mg HS tea	Yes	30 days	Significant difference noted as an increase in HDL and LDL compared to baseline	(52)
NNT	16	Female subjects with BMI > 25 kg/m^2^	2 g HS tea twice daily	No	6 weeks	Lowering the OxLDL levels of participants	(53)

Anti-inflammatory and antimicrobial properties

The flowers of HS are known for their high anthocyanin content and are widely consumed globally [54]. Anthocyanins, which are responsible for the vibrant colors in numerous fruits and vegetables commonly consumed in Western-style diets, constitute the most prevalent group of flavonoids ingested through diet.

Monocyte chemoattractant protein-1 (MCP-1) is a potent chemotactic factor for monocytes, belonging to the C-C chemokine family, and serves as a biomarker for evaluating inflammatory diseases [55]. In a study by Deshmane et al., the impact of ingesting HSE on plasma MCP-1 levels was examined in a cohort of 10 healthy volunteers. The results revealed a noteworthy decrease in MCP-1 levels at the three-hour mark (P < 0.05; 23.2%). Conversely, a randomized controlled trial involving 40 patients investigated the anti-inflammatory effects of a jelly drink, which contained a polyphenol-rich extract from Roselle calyces along with passion fruit juice and pulp (referred to as RP jelly drink), on blood levels of interleukin-10 (IL-10), IL-6, MCP-1, and tumor necrosis factor-α (TNF-α) at baseline, four weeks, and eight weeks. The findings exhibited a statistically significant reduction in TNF-α at baseline, four weeks, and eight weeks between the two groups (P = 0.026). However, no significant difference was observed among other inflammatory markers. In the investigation conducted by Joven et al., individuals diagnosed with metabolic syndrome received polyphenols derived from the calyx of HS for a duration of four weeks. The outcomes revealed notable anti-inflammatory effects, as evidenced by a reduction in interleukin IL-6, IL-1β, and IL-8. Additionally, the extract demonstrated potent antioxidant properties, manifested through a decrease in 8-isoprostane-F2α levels and an elevation in serum paraoxonase activity [56].

Studies conducted in vitro and on animal models have confirmed the antimicrobial properties of HS [57-60]. However, research on human subjects in this regard is limited. In a randomized clinical trial led by Cai et al., involving 93 females with uncomplicated urinary tract infections, it was found that twice-daily tablets containing extracts of HS and Boswellia serrata for seven days resulted in a reduction in urinary tract infection (UTI) symptoms and their recurrence, comparable to the effects of antibiotics [61]. Furthermore, Chou et al. conducted a study to investigate the potential link between consuming HS drinks and the occurrence of UTIs among residents in long-term care facilities. A survey assessing UTI prevalence was conducted on residents with urinary catheters between 2012 and 2013. The average age of the residents was approximately 78 years. Among those who did not receive an HS drink, the average annual UTI prevalence was 26.33% ± 0.98%. In contrast, those who were given HS drinks had an annual UTI incidence of 16.88% ± 2.92%. Administering HS drinks resulted in a significant 36% reduction in UTI incidence. These findings suggest that consuming HS drinks may be associated with a decreased incidence of UTIs among residents with urinary catheters in long-term care facilities. However, the study has notable limitations, including its cross-sectional design, absence of adjustments for potential confounding variables, and a lack of information regarding the diagnostic methodology used in catheterized patients [62].

A study conducted by Alshami et al. examined the antimicrobial effect of HS on uropathogens in individuals experiencing UTIs. In this study, a total of six Escherichia coli and two Klebsiella pneumoniae isolates were obtained from urine samples [63]. The susceptibility of bacterial isolates to HSE was assessed by determining their minimum inhibitory concentrations (MICs) and minimum bactericidal concentrations (MBCs). Different levels of the extracts' MIC were observed against all uropathogenic isolates, with MIC values ranging from 0.5 to 4 mg/mL, and MBC values ranging from 8 to 64 mg/mL. Both the time-kill experiment and the MBC-MIC ratio analysis demonstrated a predominantly bacteriostatic effect of the extracts. However, further studies are needed to evaluate the efficacy and safety of HS in preventing recurrent UTIs in human subjects. An investigation into the effectiveness of HS methanol extract on Gram-positive and Gram-negative bacterial cultures showed significant antibacterial activity against all the bacteria tested, particularly against Gram-positive bacteria [64]. Additionally, in a randomized controlled trial, a notable reduction in levels of high-sensitive C-reactive protein (from 3.12 to 2.52 µmol/L) was observed among patients with diabetic nephropathy after taking HS tablets twice daily for eight weeks [65].

Kidney and liver health

The positive impact of HS on renal function has been extensively examined in previous research. After an eight-week supplementation with HS pills taken twice daily, containing 425 mg of dried extract with 5.5 mg of anthocyanins, individuals with diabetic nephropathy showed improved renal function and reduced levels of blood urea nitrogen, from 33 to 24 mg/dL. Moreover, creatinine levels dropped from 1.37 to 1.09 g/dL [65]. Prasongwatana et al. conducted a study to evaluate the uricosuric effects of HS tea, administered at a dosage of 1,500 mg of HS powder in 150 mL of hot water twice daily for 15 days, on both healthy individuals and participants with a history of kidney stones. Their findings revealed increased excretion levels of oxalate, citrate, and uric acid following the consumption of HS tea in both groups. Importantly, this effect was significantly more pronounced in individuals with a history of renal stones, indicating potential long-term benefits for hyperuricemia in gout disease [66]. In a related study, Kirdpon et al. observed that HS calyx beverages did not act as a preventive measure against the formation of kidney stones. Interestingly, HS calyx beverages showed a diuretic effect in healthy young adults. The finding was dose-dependent; specifically, daily intake of HSE at a dose of 24 grams exhibited this diuretic effect. On the other hand, doses of 16 grams per day had no such diuretic effect. Nevertheless, the ingestion of HS capsules at a dosage of 500 mg per day for a period of 30 days did not lead to any observable changes in renal function among healthy volunteers aged between 18 and 45 years [67]. In a randomized, double-blind study comparing lisinopril as the control, Herrera-Arellano et al. observed that hypertensive subjects who consumed HS decoction daily for 28 days showed a slight decrease in sodium levels (from 139 to 137 mEq/L). Potassium levels remained unchanged, mirroring observations in the group receiving lisinopril [17]. In individuals aged 30-80 years with mild to moderate hypertension, a comparable trend was observed after a daily intake of HS infusion for 30 days. The infusion, comprising 10 g of HS calyx in 500 mL of water and containing 9.6 mg of anthocyanins, resulted in an increase in sodium excretion. Interestingly, this was accompanied by a tendency towards decreased chlorine levels, although there was no significant alteration in potassium excretion [5].

In a randomized controlled trial examining the impact of HS on fatty liver and liver function tests, participants were adults aged 18-65 with a BMI ≥ 27 kg/m² and diagnosed with fatty liver disease. Each HS capsule contained 450 mg of HSE and 50 mg of starch, while the placebo consisted of 500 mg of starch alone. The intervention group received two capsules of HSE three times a day for a duration of 12 weeks. The findings revealed a significant reduction in the fatty liver score among the HSE-treated subjects, decreasing by approximately 15%, from 5.21 ± 1.72 to 4.42 ± 2.01 (P = 0.018). However, no notable changes were observed in AST or ALT levels within both the HSE and control groups, and the pre-post differences between the two groups were not statistically significant [50]. The positive impacts on metabolism and metabolic regulation were linked to the polyphenols found in HS, such as flavonoids, anthocyanins, and phenolic acids. These compounds potentially operate through various metabolic pathways, including increasing PPAR-α expression and hindering hepatic lipogenesis [50].

Safety considerations

Hibiscus is generally considered a safe herbal remedy [1,3,23,68]. One study noted mild gastrointestinal symptoms with the use of 1 g of hibiscus extract in the initial week of supplementation, though these symptoms resolved within one week [49]. Nevertheless, when administered at doses of 300 mg/kg/day of HS over a period of three months, an adverse effect on liver enzymes was noted. This suggests that, at exceptionally high doses, the extract may have hepatotoxic potential [69]. Additionally, it is important to consider potential herb-drug interactions. Given that hibiscus has been identified as an ACE inhibitor [70], further research is warranted to ascertain its potential interactions with ACE inhibitors such as ramipril. Consequently, additional investigations are warranted to ensure the safety of hibiscus in conjunction with various medications.

## Conclusions

This review highlighted the diverse health benefits of HS. It showed a significant antihypertensive effect, mediated through various mechanisms. HS also showed anti-inflammatory, antimicrobial properties and potential cardiovascular benefits beyond blood pressure regulation. While some studies have reported significant reductions in blood glucose levels, as well as improvements in lipid parameters such as total cholesterol, LDL cholesterol, and triglycerides, there is some variability in the outcomes, likely influenced by factors such as dosage, duration, and formulation of HS used in the studies. HS could be a natural, cost-effective intervention for mild to moderate hypertension, diabetes, obesity, and hyperlipidemia. Further research is needed to determine optimal dosages and potential interactions with medications.
